# Supplementary material for: Food as Medicine: A Review of Plant Secondary Metabolites from Pollen, Nectar, and Resin with Health Benefits for Bees
Source: Insects. 2025 Apr 15;16(4):414. doi: 10.3390/insects16040414 (PMC12027951; doi:10.3390/insects16040414)
Supplement: Supplementary file 1 [file insects-16-00414-s001.zip › insects-3523035-supplementary.pdf]

Supplementary material for: Morrison, B., Newburn L.R. & Fitch, G., "Food as medicine: A review of plant secondary metabolites from pollen, nectar and resin with health benefits for bees."

Section S1. Literature review methods

Given the disparate literature in this field, our review occurred in two stages: 1) identifying PSMs with medicinal benefits for bees and 2) identifying plant products known to have direct medicinal effects or to contain medicinal PSMs. Beneficial resins were also included because honeybees use it to make propolis, a substance with demonstrated anti-microbial properties [1–3]. To identify PSMs with health benefits to bees, we used the Scopus database to conduct a two-stage search. First, to identify potentially beneficial PSMs, we used the following search string: (bee OR Bombus OR Apis) AND (pathogen OR pest OR parasite) AND (phytochemical OR secondary metabolite OR plant extract OR botanical). From articles that we deemed relevant, we screened citations for additional articles that the search may have missed. Then, for each PSM identified, we conducted a search pairing the PSM with (pollen OR nectar OR flower OR resin). Only papers published in English or with English-language abstracts were included. Many of the plants identified had potentially medicinal flowers, but the chemical composition of their nectar and pollen was not explicitly evaluated. As such, we excluded these species from analysis in the main text but include them in Supplementary Table S2.

Table S1. Plants with pollinator relevant products with demonstrated or potential health benefits for honeybees and bumblebees. An asterisk (*) indicates plants that were tested directly in bees or their disease agents (demonstrated effects); no asterisk indicates that the PSM(s) contained in the relevant plant product(s) has/have been shown to confer health benefits.

**Botanical family**	**Scientific name**	**Plant products**	**PSM**	**References**
Acanthaceae	*Justicia adhatoda*	Pollen	Quercetin<br>Rhamnetin	[56]
Amaranthaceae	*Chenopodium album*	Pollen	Quercetin	[57]
	Chenopodium ficifolium	Pollen	Quercetin	[57]
Amaryllidaceae	*Allium cepa*	Nectar	Luteolin	[58]
Anacardiaceae	*Pistacia lentiscus*	Resin	Carvone	[59]
Apiaceae	*Anethum graveolens*	Pollen	Kaempferol<br>Quercetin<br>Rhamnetin	[56]
Apocynaceae	*Asclepias syriaca*	Resin	Linoleic acid<br>Linolenic acid	[60]

	Gomphocarpus physocarpus	Nectar	Acetic acid	[15]
Aquifoliaceae	*Ilex serrata*	Pollen	Quercetin	[57]
Araliaceae	*Dendropanax trifidus*	Resin	Linoleic acid	[61]
	Fatsia japonica	Pollen	Quercetin	[57]
	Hedera helix	Pollen	Linoleic acid<br>Linolenic acid	[62]
Araucariaceae	**Araucaria cunninghamii*	Resin	Not identified in this review	[63]
Arecaceae	*Cocos nucifera*	Sap	Gallic acid<br>Hesperidin<br>Lauric acid<br>Myristic acid<br>Palmitoleic acid<br>p-Coumaric acid<br>Quercetin<br>Rutin	[64,65]
	Elaeis guineensis	Pollen	Linoleic acid<br>Linolenic acid	[62,66]
	Phoenix dactylifera	Pollen	Gallic acid<br>Kaempferol<br>Linoelaidic acid<br>Linoleic acid<br>Linolenic acid<br>Luteolin<br>Palmitoleic acid<br>p-Coumaric acid<br>Quercetin<br>Rhamnetin<br>Rutin	[67–73]

	Raphia hookeri	Pollen	Linoleic acid<br>Myristic acid	[62]
	Raphia regalis	Pollen	Linoleic acid<br>Linolenic acid	[62]
	Raphia vinifera	Pollen	Linoleic acid	[62]
	Trachycarpus fortune	Pollen	Quercetin<br>Rhamnetin	[57]
Asphodelaceae	*Xanthorrhoea preisii*	Pollen	Linoleic acid<br>Linolenic acid	[62]
Asteraceae	**Helianthus annuus*	Pollen	Kaempferol<br>Lauric acid<br>Linoleic acid<br>Linolenic acid<br>Myristic acid	[57,74–84]
	**Solidago* spp.	Pollen	Not identified in this review	[76]
	Achillea millefolium	Pollen	Quercetin	[85]
	Anacyclus clavatus	Pollen	Luteolin	[56]
	Anacyclus radiatus	Pollen	Luteolin<br>Quercetin	[56]
	Arcotheca calendula	Pollen	Linoleic acid	[62]
	Artemisia spp.	Pollen	Quercetin	[57]
	Aster novi-belgii	Pollen	Linoleic acid<br>Linolenic acid	[62]
	Calendula officinalis	Pollen	Quercetin<br>Rhamnetin	[56]
	Centaurea cyanus	Pollen	Quercetin	[57]
	Cosmos bipinnatus	Pollen	Quercetin	[57]
	Elytropappus rhinocerotis	Resin	Luteolin<br>Quercetin	[86]

	Glebionis coronaria	Pollen	Luteolin<br>Quercetin	[85]
	Helianthus tuberosus	Pollen	Kaempferol	[57]
	Helminthotheca echioides	Pollen	Linoleic acid<br>Linolenic acid	[62]
	Tanacetum vulgare	Pollen	Quercetin	[85]
	Taraxacum spp.	Pollen	Cinnamic acid<br>Linoleic acid<br>Linolenic acid	[62,87]
	Urospermum dalechampii	Pollen	Linoleic acid<br>Linolenic acid	[62]
Betulaceae	*Alnus cordata*	Pollen	Quercetin	[88]
	Alnus glutinosa	Pollen	p-Coumaric acid	[89]
	Alnus japonica	Pollen	Quercetin<br>Rhamnetin	[57]
	Betula pendula	Resin	Quercetin	[90]
	Carpinus spp.	Pollen	Quercetin	[57]
	Corylus avellana	Pollen	Quercetin<br>Kaempferol	[91]
	Corylus heterophylla	Pollen	Quercetin	[57]
	Corylus sieboldiana	Pollen	Quercetin	[57]
Bignoniaceae	*Campsis grandiflora*	Pollen	Kaempferol	[57]
Boraginaceae	*Echium creticum*	Pollen	Kaempferol	[56]
Brassicaceae	*Brassica* spp.	Pollen	Cinnamic acid<br>Linolenic acid<br>Myristic acid<br>Abscisic acid	[62,87,92–97]

			Eicosenoic acid<br>Indole-3-acetic acid<br>Kaempferol<br>Lauric acid<br>Linoleic acid<br>Luteolin<br>Naringenin<br>Palmitoleic acid<br>Quercetin	
Burseraceae	*Boswellia ameero*	Resin	Terpineol	[98]
	Boswellia frereana	Resin	Terpineol	[99]
	Boswellia serrata	Resin	Sabinene<br>Terpineol	[100,101]
	Commimorpha myrrh	Resin	Caffeine<br>Cinnamic acid<br>Gallic acid<br>Naringenin<br>Quercetin<br>Rutin	[102]
	Pachylobus edulis	Resin	Sabinene<br>Terpineol	[103]
	Protium spp.	Resin	Terpineol<br>Sabinene<br>Thymol	[104–107]
Cactaceae	*Opuntia ficus-indica*	Pollen	Kaempferol<br>Rhamnetin	[56]
Campanulaceae	*Adenophora triphylla*	Pollen	Quercetin	[57]
	Platycodon grandiflora	Pollen	Quercetin	[57]

Cannabaceae	*Cannabis sativa*	Pollen	Kaempferol<br>Linoleic acid<br>Linolenic acid<br>Quercetin	[57,108,109]
	Humulus scandens	Pollen	Kaempferol	[57]
Capparaceae	*Capparis spinosa*	Pollen	Kaempferol<br>Quercetin	[56]
Caprifoliaceae	*Scabiosa* spp.	Pollen	Linolenic acid	[62]
	Weigela spp.	Pollen	Quercetin	[57]
Clethraceae	*Clethra barbinervis*	Pollen	Kaempferol<br>Quercetin	[57]
Cornaceae	*Cornus* spp.	Pollen	Quercetin	[57]
Cucurbitaceae	*Bryonia dioica*	Pollen	Kaempferol	[110]
	Citrullis lanatus	Pollen	Kaempferol	[110]
	Cucurbita maxima	Pollen	beta-Carotene	(Togasawa et al. 1967)
	Lagenaria siceraria	Pollen	Kaempferol	[110]
	Sicyos edulis	Pollen	Kaempferol	[110]
Cupressaceae	*Biota orientalis*	Pollen	Luteolin<br>p-Coumaric<br>Quercetin	[111]
	Cryptomerica japonica	Pollen	Luteolin<br>Naringenin	[110]
Cycadaceae	*Macrozamia reidlei*	Pollen	Linolenic acid	[62]
Cytinaceae	*Cytinus hypocistis*	Nectar	Linoleic acid<br>Linolenic acid<br>Linoelaidic acid	[112]

Ericaceae	**Calluna* spp.	Nectar<br>Pollen	Kaempferol<br>Quercetin<br>Rhamnetin<br>Callunene	[113–115]
	Arbutus unedo	Nectar	Abscisic acid	[116]
	Erica spp.	Nectar	Abscisic acid	[117]
	Styphelia conostephioides	Pollen	Linoleic acid<br>Linolenic acid	[62]
	Vaccinium corymbosum	Pollen	Caffeoylquinic acid<br>Kaempferol<br>Quercetin<br>Rhamnetin<br>Rutin	[118]
Eucommiaceae	*Eucommia ulmoides*	Pollen	Aucubin<br>Kaempferol<br>Naringenin<br>p-Coumaric acid<br>Quercetin<br>Rutin	[119]
Fabaceae	*Acacia pycantha*	Pollen	Quercetin	[56]
	Melilotus messanensis	Pollen	Myristic acid	[62]
	Onobrychis viciifolia	Pollen<br>Nectar	Caffeine<br>Linoleic acid<br>Linolenic acid	[62,120]
	Trifolium spp.	Pollen	Cinnamic acid<br>Linolenic acid<br>Myristic acid	[62,87]
	Vicia spp.	Pollen	Cinnamic acid<br>Linolenic acid	[62,87]

			Myristic acid	
Fagaceae	*Quercus acutissima*	Pollen	Quercetin<br>Rhamnetin	[57]
	Quercus serrata	Pollen	Quercetin<br>Rhamnetin	[57]
Gelsemiaceae	*Gelsemium sempervirens*	Nectar	Gelsemine	[121]
Gingkoaceae	*Gingko biloba*	Pollen	Kaempferol	[122]
Hypericaceae	*Hypericum maculatum*	Pollen	Quercetin	[85]
Iridaceae	*Gladiolus x gandavensis*	Pollen	Kaempferol<br>Quercetin	[57]
Juglandaceae	*Juglans ailantifolia*	Pollen	Quercetin	[57,88]
	Juglans regia	Pollen	Quercetin<br>Kaempferol	[123]
	Pterocarya fraxinifolia	Pollen	p-Coumaric acid	[89]
Lamiaceae	*Mentha spicata*	Pollen	Luteolin	[56]
	Monarda fistulosa	Nectar<br>Pollen	Carvacrol<br>Thymol	Fitch et al. *unpublished*
	Thymus vulgaris	Nectar<br>Pollen	Carvacrol<br>Geraniol<br>Sabinene<br>Terpineol<br>Thymol	[124]
Liliaceae	*Lilium* spp.	Pollen	Quercetin<br>Rhamnetin<br>beta-Carotene<br>Rutin	[44,57,125,126]

	Tulipa spp.	Pollen	Quercetin	[127]
Linaceae	*Linum usitassimum*	Pollen	Linolenic acid<br>Myristic acid	[62]
Magnoliaceae	*Magnolia* spp.	Pollen	Rhamnetin	[57]
Malvaceae	*Hibiscus mutabilis*	Pollen	Kaempferol	[57]
	Hibiscus syriacus	Pollen	Kaempferol	[57]
	Malva sylvestris	Pollen	Quercetin	[56]
	Malva verticillata	Pollen	Quercetin	[57]
	Tilia spp.	Nectar	Caffeine<br>Kaempferol<br>Quercetin<br>Rhamnetin<br>Rutin	[128–130]
Moraceae	*Ficus dubia*	Sap	Caffeoylquinic acid	[131]
Myrtaceae	**Corymbia torelliana*	Resin	Not identified in this review	[63]
	Corymbia calophylla	Pollen	Linoleic acid<br>Linolenic acid	[62]
	Eucalyptus spp.	Pollen<br>Resin	Cinnamic acid<br>Gallic acid<br>Kaempferol<br>p-Coumaric acid<br>Linoleic acid	[62,132–134]
Nothofagaceae	*Nothofagus antarctica*	Pollen	Kaempferol<br>Quercetin	[135]
Oleaceae	*Forsythia* spp.	Pollen	Quercetin<br>Rutin	[57,136]
	Ligustrum spp.	Pollen	Kaempferol	[57]

	Olea europea	Pollen	Indole-3-acetic acid	[137]
	Osmanthus heterophyllus	Pollen	Quercetin	[57]
Orchidaceae	*Dendrobium* spp.	Pollen	Abscisic acid<br>Indole-3-acetic acid	[138]
	Ophrys spp.	Pollen	Kaempferol	[139]
Papaveraceae	*Papaver rhoeas*	Pollen	Kaempferol<br>Linolenic acid<br>Luteolin	[56,62]
	Papaver somniferum	Pollen	Quercetin	[57]
Pinaceae	**Pinus* spp.	Pollen<br>Resin	p-Coumaric acid<br>Linoleic acid<br>Linolenic acid<br>Abscisic acid<br>Sabinene<br>Terpineol	[62,63,140–144]
	Cedrus deodora	Pollen	Naringenin	[145]
	Pseudostuga macrocarpa	Pollen	Linoleic acid<br>Linolenic acid	[140]
	Pseudostuga menziesii	Pollen	Linoleic acid	[62,140]
	Pseudostuga wilsoniana	Pollen	Linoleic acid	[62,140]
Plantaginaceae	*Antirrhinum* spp.	Pollen	Linolenic acid	[62]
	Chelone glabra	Pollen<br>Nectar	Aucubin<br>Catalpol	[146]
Poaceae	*Zea mays*	Pollen	beta-Carotene	[57,62,126,147–150]

			Lauric acid<br>Linoelaidic acid<br>Linoleic acid<br>Linolenic acid<br>Myristic acid<br>Palmitoleic acid<br>Quercetin<br>Rhamnetin<br>Rutin<br>Tridecanoic acid<br>Undecanoic acid	
Polygonaceae	*Fagopyrum esculentum*	Pollen<br>Nectar	Caffeoylquinic acid<br>Cinnamic acid<br>Gallic acid<br>Kaempferol<br>Luteolin<br>p-Coumaric acid<br>Quercetin<br>Pinocembrin<br>Pinobanksin<br>Rhamnetin<br>Resveratrol<br>Rutin	[151,152]
Putranjivaceae	*Putranjiva roxburghii*	Pollen	Cinnamic acid<br>Gallic acid<br>Kaempferol<br>p-Coumaric acid	[153]
Rosaceae	*Crataegus cuneata*	Pollen	Quercetin	[57]
	Filipendula spp.	Pollen	Cinnamic acid	[87]
	Prunus spp.	Nectar<br>Pollen	Amygdalin<br>Kaempferol	[62,90,154–158]

		Resin	Linoleic acid<br>Linolenic acid<br>p-Coumaric acid<br>Rutin<br>Naringenin<br>Quercetin<br>Cinnamic acid<br>Hesperidin<br>Rhamnetin	
	Rosa spp.	Pollen	Abscisic acid	[159]
	Rubus spp.	Pollen	Quercetin<br>Cinnamic acid	[57,87]
Rubiaceae	*Coffea* spp.	Nectar	Caffeine	[160]
Rutaceae	*Citrus* spp.	Nectar<br>Pollen	Caffeine<br>Linoleic acid<br>Linolenic acid<br>Myristic acid<br>Hexenal	[62,160–163]
Salicaceae	**Salix* spp.	Pollen	Quercetin	[57,164,165]
	Populus spp.	Resin	p-Coumaric acid<br>Pinocembrin<br>Quercetin<br>Cinnamic acid<br>Kaempferol<br>Naringenin<br>Pinobanksin	[90,166]
Sapindaceae	*Acer saccharum*	Sap	Acetic acid	[167]
Solanaceae	*Nicotiana* spp.	Nectar<br>Pollen	Nicotine<br>Anabasine	[168–172]

	Solandra grandiflora	Pollen	Linoleic acid<br>Linolenic acid	[62]
	Solanum lycopersium	Pollen	Abscisic acid<br>Indole-3-acetic acid	[93]
Strelitziaceae	*Strelitzia reginae*	Pollen	Linoleic acid<br>Linolenic acid	[62]
Styracaceae	*Styrax bensoin*	Resin	Cinnamic acid	[173]
Tropaeolaceae	*Tropaelum majus*	Pollen	beta-Carotene	[174]
Typhaceae	*Typha* spp.	Pollen	Quercetin<br>Rhamnetin<br>Kaempferol<br>Naringenin<br>Linoleic acid<br>Linolenic acid	[57,175–179]
Viburnaceae	*Viburnum* spp.	Pollen	Quercetin	[57]

Table S2. Plants whose flowers (but not necessarily floral products) either A) have beneficial effects on honeybees or bumblebees or B) contain PSMs with beneficial impacts. In part B, an asterisk (*) indicates plants that were tested directly in bees or their disease agents.

**A) Flowers whose extracts have beneficial effects**			
**Family**	**Species**	**Health benefit**	**References**
Apiaceae	*Anethum graveolens*	Defense (wax moth)	[4]
Apiaceae	*Coriandrum sativum*	Defense (wax moth)	[4]
Asteraceae	*Achyrocline saturejoides*	Defense (foulbrood)	[5,6]
Asteraceae	*Artemisia absinthium*	Defense (foulbrood)	[7]

Asteraceae	*Artemisia annua*	Defense (foulbrood)	[7]
Asteraceae	*Baccharis aliena*	Defense (chalkbrood, *Varroa*)	[8]
Asteraceae	*Baccharis flabellata*	Defense (*Varroa*)	[9]
Asteraceae	*Pseudognaphalium gaudichaudianum*	Defense (foulbrood)	[5]
Asteraceae	*Solidago chilensis*	Defense (foulbrood)	[10]
Asteraceae	*Tagetes minuta*	Defense (foulbrood, *Varroa*)	[5,11,12]
Astercaeae	*Calendula officinalis*	Defense (foulbrood)	[13]
Lamiaceae	*Lepechinia floribunda*	Defense (foulbrood)	[7]
Lamiaceae	*Marrubium vulgare*	Defense (foulbrood)	[5]
Lamiaceae	*Minthostachys verticillata*	Defense (foulbrood, *Varroa*)	[5,9]
Lamiaceae	*Minthostachys mollis*	Defense (foulbrood)	[10]
Lamiaceae	*Salvia rosmarinus*	Defense (foulbrood)	[14]
Myrtaceae	*Eucalyptus cinerea*	Defense (foulbrood)	[5]
**B) Flowers containing beneficial PSMs**			
**Family**	**Species**	**PSM**	**References**
Anacardiaceae	*Schinus molle*	Carvacrol	[10]

Apocynaceae	*Gomphocarpus fructicosus*	Eugenol	[15]
Asparagaceae	*Agave durangensis*	Kaempferol<br>Quercetin	[16]
Asteraceae	*Artemisia fragrans*	Carvacrol<br>Carvone<br>Eugenol<br>Terpineol	[17]
Asteraceae	*Aster indicus*	Caffeoylquinic acid<br>Rutin	[18]
Asteraceae	*Carthamus eriocephalus*	Kaempferol	[19]
Asteraceae	*Carthamus tinctorius*	Caffeoylquinic acid<br>p-Coumaric acid	[20]
Asteraceae	*Coreopsis tinctorius*	Luteolin<br>Quercetin	[21]
Asteraceae	*Echinops spinosissimus*	Cinnamic acid<br>Gallic acid<br>Kaempferol<br>p-Coumaric acid<br>Quercetin<br>Rutin	[22]
Asteraceae	*Flaveria bidentis*	Rhamnetin	[23]
Begoniaceae	*Begonia cucullata*	Quercetin	[24]
Cannabaceae	**Humulus lupulus*	Geraniol	[25]
Clusiaceae	*Garcinia indica*	Kaempferol<br>Luteolin	[26]

		Quercetin	
Ericaceae	*Rhododendron ponticum*	p-Coumaric acid	[27]
Fabaceae	*Arachis hypogaea*	p-Coumaric acid<br>Quercetin<br>Rhamnetin	[28]
Fabaceae	*Astragalus membranaceus*	Kaempferol<br>Quercetin<br>Rhamnetin	[29]
Fabaceae	*Wisteria floribunda*	Kaempferol<br>Luteolin<br>Quercetin	[30]
Fagaceae	*Fagus sylvatica*	Kaempferol	[31]
Hypericaceae	*Hypericum spp.*	Epiclusianone<br>Hyperforin<br>Uligosin	[32]
Lamiaceae	**Origanum vulgare*	Terpineol<br>Thymol<br>Carvacrol	[33,34]
Lamiaceae	**Sartureia hortensis*	Carvacrol	[33]
Lamiaceae	*Elsholtzia splendens*	Luteolin	[35]
Lamiaceae	*Marrubium fridwaldskynum*	Carvacrol	[36]
Lamiaceae	*Ocimum basilicum*	Eugenol	[33]
Lamiaceae	*Ocimum carnosum*	Eugenol	[37]
Lamiaceae	*Origanum elongatum*	Carvacrol<br>Thymol	[38]
Lamiaceae	*Salvia verticillata*	Terpineol	[39,40]

Lamiaceae	*Thymus saturejoides*	Carvacrol<br>Terpineol<br>Thymol	[38]
Lauraceae	**Cryptocarya alba*	Terpineol	[41]
Lauraceae	*Laurus nobilis*	Eugenol<br>Sabinene<br>Terpineol	[42]
Liliaceae	*Gagea taurica*	Hesperidin	[43]
Liliaceae	*Lilium longiflorum*	Kaempferol<br>Quercetin	[44]
Liliaceae	*Tulipa gesneriana*	Delphinidin	[45]
Myrtaceae	*Syzygium aromaticum*	Eugenol	[39]
Pittosporaceae	*Pittosporum tobira*	Kaempferol<br>Quercetin	[30]
Poaceae	*Cymbopogan martinii*	Geraniol	[46]
Polemoniaceae	*Collomia* sp.	Kaempferol<br>Quercetin<br>Delphinidin	[47]
Polygonaceae	**Bistorta officinalis*	Terpineol	[48]
Primulaceae	*Cyclamen purpurascens*	Delphinidin	[49]
Saliaceae	*Salix caprea*	Rhamnetin	[50]
Schisandraceae	*Schisandra chinensis*	Cinnamaldehyde<br>Eugenol<br>Linoleic acid	[51]
Scrophulariaceae	*Buddleja globosa*	Terpineol	[10]
Theaceae	*Camellia nitidissima*	Kaempferol	[52]

		Quercetin	
Verbenaceae	*Lantana camara*	Beta-carotene<br>Delphinidin	[53]
Verbenaceae	**Acantholippia seriphioides*	Carvacrol<br>Thymol<br>Carvone	[10,12,54,55]
Verbenaceae	**Aloysia polystachia*	Carvone	[7,10]
Verbenaceae	**Lippia turbinata*	Sabinene<br>Carvone<br>Terpineol	[5,10,54]

References

1. Simone-Finstrom, M.; Borba, R.S.; Wilson, M.; Spivak, M. Propolis Counteracts Some Threats to Honey Bee Health. *Insects* **2017**, *8*, 1–20, doi:10.3390/insects8020046.

2. Niu, G.; Johnson, R.M.; Berenbaum, M.R. Toxicity of Mycotoxins to Honeybees and Its Amelioration by Propolis. *Apidologie* **2011**, *42*, 79–87, doi:10.1051/apido/2010039.

3. Simone, M.; Evans, J.D.; Spivak, M. Resin Collection and Social Immunity in Honey Bees. *Evolution (N Y)* **2009**, *63*, 3016–3022, doi:10.1111/j.1558-5646.2009.00772.x.

4. Oulebsir-Mohandkaci, H.; Baba Aissa, A.; Badaoui, S.; Bouyahiaoui, H.; Ait Kaki, S.; Mohammedi, A. Comparative Study of the Toxicity of Phenolic Compounds of Coriander (*Coriandrum Sativum*) and False Fennel (*Aneth Graveolens*) on *Galleria Mellonella* (Lepidoptera, Pyralidae). *EuroMediterr J Environ Integr* **2018**, *3*, 1–7, doi:10.1007/s41207-018-0071-z.

5. González, M.J.; Marioli, J.M. Antibacterial Activity of Water Extracts and Essential Oils of Various Aromatic Plants against *Paenibacillus Larvae*, the Causative Agent of American Foulbrood. *J Invertebr Pathol* **2010**, *104*, 209–213, doi:10.1016/j.jip.2010.04.005.

6. González, M.J.; Beoletto, V.G.; Agnese, A.M.; Audisio, M.C.; Marioli, J.M. Purification of Substances from *Achyrocline Satureioides* with Inhibitory Activity Against *Paenibacillus Larvae*, the Causal Agent of American Foulbrood in Honeybees' Larvae. *Appl Biochem Biotechnol* **2015**, *175*, 3349–3359, doi:10.1007/s12010-015-1506-5.

7. Fuselli, S.R.; Garcia de la Rosa, S.B.; Eguaras, M.J.; Fritz, R. Susceptibility of the Honeybee Bacterial Pathogen *Paenibacillus Larvae* to Essential Oils Distilled from Exotic and Indigenous Argentinean Plants. *Journal of Essential Oil Research* **2008**, *20*, 464–470, doi:10.1080/10412905.2008.9700060.

8. Ruffinengo, S.R.; Maggi, M.; Fuselli, S.; Floris, I.; Clemente, G.; Firpo, N.H.; Bailac, P.N.; Ponzi, M.I. Laboratory Evaluation of Heterothalamus Alienus Essential Oil Against Different Pests of Apis Mellifera. *Journal of Essential Oil Research* **2006**, *18*, 704–707, doi:10.1080/10412905.2006.9699211.

9. Damiani, N.; Gende, L.B.; Maggi, M.D.; Palacios, S.; Marcangeli, J.A.; Eguaras, M.J. Repellent and Acaricidal Effects of Botanical Extracts on Varroa Destructor. *Parasitol Res* **2011**, *108*, 79–86, doi:10.1007/s00436-010-2043-3.

10. Pellegrini, M.C.; Alonso-Salces, R.M.; Umpierrez, M.L.; Rossini, C.; Fuselli, S.R. Chemical Composition, Antimicrobial Activity, and Mode of Action of Essential Oils against *Paenibacillus Larvae*, Etiological Agent of American Foulbrood on *Apis Mellifera*. *Chem Biodivers* **2017**, *14*, 1–18, doi:10.1002/cbdv.201600382.

11. Eguaras, M.J.; Fuselli, S.; Gende, L.; Fritz, R.; Ruffinengo, S.R.; Clemente, G.; Gonzalez, A.; Bailac, P.N.; Ponzi, M.I. An in Vitro Evaluation of *Tagetes Minuta* Essential Oil for the Control of the Honeybee Pathogens *Paenibacillus Larvae* and *Ascosphaera Apis*, and the Parasitic Mite *Varroa Destructor*. *Journal of Essential Oil Research* **2005**, *17*, 336–340, doi:10.1080/10412905.2005.9698924.

12. Fuselli, S.R.; Gende, L.B.; Garcia de la Rosa, S.B.; Eguaras, M.J.; Fritz, R. Short Communication. Inhibition of *Paenibacillus Larvae* Subsp. *Larvae* by the Essential Oils of Two Wild Plants and Their Emulsifying Agents. *Spanish Journal of Agricultural Research* **2005**, *3*, 220–224, doi:10.5424/sjar/2005032-147.

13. Piana, M.; de Brum, T.F.; Boligon, A.A.; Alves, C.F.S.; de Freitas, R.B.; Nunes, L.T.; Mossmann, N.J.; Janovik, V.; Jesus, R.S.; Vaucher, R.A.; et al. In Vitro Growth-Inhibitory Effect of Brazilian Plants Extracts against *Paenibacillus Larvae* and Toxicity in Bees. *Annals of the Brazilian Academy of Sciences* **2015**, *87*, 1041–1047, doi:10.1590/0001-3765201520140282.

14. Flesar, J.; Havlik, J.; Kloucek, P.; Rada, V.; Titera, D.; Bednar, M.; Stropnicky, M.; Kokoska, L. In Vitro Growth-Inhibitory Effect of Plant-Derived Extracts and Compounds against *Paenibacillus Larvae* and Their Acute Oral Toxicity to Adult Honey Bees. *Vet Microbiol* **2010**, *145*, 129–133, doi:10.1016/j.vetmic.2010.03.018.

15. Burger, H.; Buttala, S.; Koch, H.; Ayasse, M.; Johnson, S.D.; Stevenson, P.C. Nectar Cardenolides and Floral Volatiles Mediate a Specialized Wasp Pollination System. *Journal of Experimental Biology* **2024**, *227*, 1–12, doi:10.1242/jeb.246156.

16. Barriada-Bernal, L.G.; Almaraz-Abarca, N.; Delgado-Alvarado, E.A.; Gallardo-Velázquez, T.; Ávila-Reyes, J.A.; Torres-Morán, M.I.; del Socorro González-Elizondo, M.; Herrera-Arrieta, Y. Flavonoid Composition and Antioxidant Capacity of the Edible Flowers of *Agave Durangensis* (Agavaceae). *CyTA - Journal of Food* **2014**, *12*, 105–114, doi:10.1080/19476337.2013.801037.

17. Movafeghi, A.; Djozan, Dj.; Torbati, S. Solid-Phase Microextraction of Volatile Organic Compounds Released from Leaves and Flowers of *Artemisia Fragrans*, Followed by GC and GC/MS Analysis. *Nat Prod Res* **2010**, *24*, 1235–1242, doi:10.1080/14786410903108951.

18. Zhong, R.-F.; Xu, G.-B.; Wang, Z.; Wang, A.-M.; Guan, H.-Y.; Li, J.; He, X.; Liu, J.-H.; Zhou, M.; Li, Y.-J.; et al. Identification of Anti-Inflammatory Constituents from *Kalimeris Indica* with UHPLC-ESI-Q-TOF-MS/MS and GC-MS. *J Ethnopharmacol* **2015**, *165*, 39–45, doi:10.1016/j.jep.2015.02.034.

19. Shabana, M.M.; El-Sherei, M.M.; Moussa, M.Y.; Sleem, A.A.; Abdallah, H.M. Investigation of Phenolic Constituents of *Carduncellus Eriocephalus* Boiss. Var. *Albiflora* Gauba and Their Biological Activities. *Nat Prod Commun* **2007**, *2*, 823–828.

20. Li, L.; Yang, Y.; Hou, X.; Gu, D.; Ba, H.; Abdulla, R.; Wu, G.; Xin, X.; Aisa, H.A. Bioassay-Guided Separation and Purification of Water-Soluble Antioxidants from *Carthamus Tinctorius* L. by Combination of Chromatographic Techniques. *Sep Purif Technol* **2013**, *104*, 200–207, doi:10.1016/j.seppur.2012.11.027.

21. Sun, Y.-H.; Zhao, J.; Jin, H.-T.; Cao, Y.; Ming, T.; Zhang, L.-L.; Hu, M.-Y.; Hamlati, H.; Pang, S.-B.; Ma, X.-P. Vasorelaxant Effects of the Extracts and Some Flavonoids from the Buds of *Coreopsis Tinctoria*. *Pharm Biol* **2013**, *51*, 1158–1164, doi:10.3109/13880209.2013.782320.

22. Al Masoudi, L.M.; Hashim, A.M. Morphological Features and Biological Activity of Different Extracts of *Echinops Spinosissimus* Grown in Saudi Arabia. *Agronomy* **2023**, *13*, 1–15, doi:10.3390/agronomy13020573.

23. Xie, Q.; Yin, L.; Zhang, G.; Wei, Y. Separation and Purification of Isorhamnetin 3-Sulphate from *Flaveria Bidentis* (L.) Kuntze by Counter-Current Chromatography Comparing Two Kinds of Solvent Systems. *J Sep Sci* **2012**, *35*, 159–165, doi:10.1002/jssc.201100554.

24. Kwon, J.-H.; Oh, H.-J.; Lee, D.-S.; In, S.-J.; Seo, K.-H.; Jung, J.-W.; Cha, B.-J.; Lee, D.Y.; Baek, N.-I. Pharmacological Activity and Quantitative Analysis of Flavonoids Isolated from the Flowers of *Begonia Semperflorens* Link et Otto. *Appl Biol Chem* **2019**, *62*, 1–8, doi:10.1186/s13765-019-0416-6.

25. Iglesias, A.; Mitton, G.; Szawarski, N.; Cooley, H.; Ramos, F.; Meroi Arcerito, F.; Brasesco, C.; Ramirez, C.; Gende, L.; Eguaras, M.; et al. Essential Oils from *Humulus Lupulus* as Novel Control Agents against *Varroa Destructor*. *Ind Crops Prod* **2020**, *158*, 1–7, doi:10.1016/j.indcrop.2020.113043.

26. Patil, R. V.; Apine, O.A.; Pawar, K.D. Comparative Flower Metabolomics Analysis in Polygamodioecious *Garcinia Indica* Choisy Indicates Flower Gender Type Specific Metabolite Accumulation. *Biocatal Agric Biotechnol* **2020**, *30*, doi:10.1016/j.bcab.2020.101836.

27. Malkoç, M.; Laghari, A.Q.; Kolayli, S.; Can, Z. Phenolic Composition and Antioxidant Properties of *Rhododendron Ponticum*: Traditional Nectar Source for Mad Honey. *Analytical Chemistry Letters* **2016**, *6*, 224–231, doi:10.1080/22297928.2016.1196605.

28. Sobolev, V.S.; Sy, A.A.; Gloer, J.B. Spermidine and Flavonoid Conjugates from Peanut (*Arachis Hypogaea*) Flowers. *J Agric Food Chem* **2008**, *56*, 2960–2969, doi:10.1021/jf703652a.

29. Li, Y.; Guo, S.; Zhu, Y.; Yan, H.; Qian, D.; Wang, H.; Yu, J.; Duan, J. Flowers of *Astragalus Membranaceus* Var. *Mongholicus* as a Novel High Potential by-Product: Phytochemical Characterization and Antioxidant Activity. *Molecules* **2019**, *24*, 1–17, doi:10.3390/molecules24030434.

30. Ono, M.; Iwashina, T. Quantitative Flavonoid Variation Accompanied by Change of Flower Colors in *Edgeworthia Chrysantha*, *Pittosporum Tobira* and *Wisteria Floribunda*. *Nat Prod Commun* **2015**, *10*, 413–416.

31. Tissut, M. Étude Spectrophotométrique et Chromatograhique Des Flavonols Du Hêtre (*Fagus Sylvatica* L.). *Phytochemistry* **1967**, *6*, 1291–1296.

32. Hernández-López, J.; Crockett, S.; Kunert, O.; Hammer, E.; Schuehly, W.; Bauer, R.; Crailsheim, K.; Riessberger-Gallé, U. In Vitro Growth Inhibition by *Hypericum* Extracts and Isolated Pure Compounds of *Paenibacillus Larvae*, a Lethal Disease Affecting Honeybees Worldwide. *Chem Biodivers* **2014**, *11*, 695–708, doi:10.1002/cbdv.201300399.

33. Albo, G.N.; Henning, C.; Ringuelet, J.; Reynaldi, F.J.; De Giusti, M.R.; Alippi, A.M. Evaluation of Some Essential Oils for the Control and Prevention of American Foulbrood Disease in Honey Bees. *Apidologie* **2003**, *34*, 417–427, doi:10.1051/apido:2003040.

34. Morshedloo, M.R.; Salami, S.A.; Nazeri, V.; Maggi, F.; Craker, L. Essential Oil Profile of Oregano (*Origanum Vulgare* L.) Populations Grown under Similar Soil and Climate Conditions. *Ind Crops Prod* **2018**, *119*, 183–190, doi:10.1016/j.indcrop.2018.03.049.

35. Peng, H.; Xing, Y.; Gao, L.; Zhang, L.; Zhang, G. Simultaneous Separation of Apigenin, Luteolin and Rosmarinic Acid from the Aerial Parts of the Copper-Tolerant Plant *Elsholtzia Splendens*. *Environmental Science and Pollution Research* **2014**, *21*, 8124–8132, doi:10.1007/s11356-014-2747-5.

36. Zheljazkov, V.D.; Semerdjieva, I.B.; Stevens, J.F.; Wu, W.; Cantrell, C.L.; Yankova-Tsvetkova, E.; Koleva-Valkova, L.H.; Stoyanova, A.; Astatkie, T. Phytochemical Investigation and Reproductive Capacity of the Bulgarian Endemic Plant Species *Marrubium Friwaldskyanum* Boiss. (Lamiaceae). *Plants* **2022**, *11*, 1–21, doi:10.3390/plants11010114.

37. Martins, E.R.; Casali, V.W.D.; Barbosa, L.C.A.; Carazza, F. Essential Oil in the Taxonomy of *Ocimum Selloi* Benth. *J Braz Chem Soc* **1997**, *8*, 29–32.

38. Ramzi, H.; Ismaili, M.R.; Aberchane, M.; Zaanoun, S. Chemical Characterization and Acaricidal Activity of *Thymus Satureioides* C. & B. and *Origanum Elongatum* E. & M. (Lamiaceae) Essential Oils against *Varroa Destructor* Anderson & Trueman (Acari: Varroidae). *Ind Crops Prod* **2017**, *108*, 201–207, doi:10.1016/j.indcrop.2017.06.031.

39. Maggi, M.D.; Ruffinengo, S.R.; Gende, L.B.; Sarlo, E.G.; Eguaras, M.J.; Bailac, P.N.; Ponzi, M.I. Laboratory Evaluations of *Syzygium Aromaticum* (L.) Merr. et Perry Essential Oil against *Varroa Destructor*. *Journal of Essential Oil Research* **2010**, *22*, 119–122, doi:10.1080/10412905.2010.9700278.

40. Giuliani, C.; Ascrizzi, R.; Lupi, D.; Tassera, G.; Santagostini, L.; Giovanetti, M.; Flamini, G.; Fico, G. *Salvia Verticillata*: Linking Glandular Trichomes, Volatiles and Pollinators. *Phytochemistry* **2018**, *155*, 53–60, doi:10.1016/j.phytochem.2018.07.016.

41. Bravo, J.; Carbonell, V.; Sepúlveda, B.; Delporte, C.; Valdovinos, C.E.; Martín-Hernández, R.; Higes, M. Antifungal Activity of the Essential Oil Obtained from *Cryptocarya Alba* against Infection in Honey Bees by *Nosema Ceranae*. *J Invertebr Pathol* **2017**, *149*, 141–147, doi:10.1016/j.jip.2017.08.012.

42. Flamini, G.; Cioni, P.L.; Morelli, I. Differences in the Fragrances of Pollen and Different Floral Parts of Male and Female Flowers of *Laurus Nobilis*. *J Agric Food Chem* **2002**, *50*, 4647–4652, doi:10.1021/jf020269x.

43. Yuca, H.; Karakaya, S.; Ekşi, G.; Aydin, B.; Goger, G.; Bona, M.; Tekman, E.; Şahin, A.A.; Sytar, O.; Pinar, N.M.; et al. Anatomical, Morphological, and Chemical Characterizations and Biological Activities of *Gagea Taurica* Steven (Liliaceae): A New Record for the Turkish Flora. *Chem Biodivers* **2023**, *20*, 1–14, doi:10.1002/cbdv.202300416.

44. Francis, J.A.; Rumbeiha, W.; Nair, M.G. Constituents in Easter Lily Flowers with Medicinal Activity. *Life Sci* **2004**, *76*, 671–683, doi:10.1016/j.lfs.2004.10.001.

45. Nakayama, M.; Okada, M.; Taya-Kizu, M.; Urashima, O.; Kan, Y.; Fukui, Y.; Koshioka, M. *Coloration and Anthocyanin Profile in Tulip Flowers*; 2004; Vol. 38;.

46. Kumar, A.; Gautam, R.D.; Kumar, R.; Chauhan, R.; Kumar, M.; Singh, S.; Kumar, D.; Singh, S.; Kumar, A. Floral Studies of Palmarosa [*Cymbopogon Martinii* (Roxb.) W. Watson] and Chemical Insights during Inflorescence Development. *Ind Crops Prod* **2021**, *171*, 1–13, doi:10.1016/j.indcrop.2021.113960.

47. Wilken, D.H.; Smith, D.M.; Harborne, J.B.; Glennie, C.W. Flavonoid and Anthocyanin Patterns and the Systematic Relationships in *Collomia*. *Biochem Syst Ecol* **1982**, *10*, 239–243.

48. Cecotti, R.; Carpana, E.; Falchero, L.; Paoletti, R.; Tava, A. Determination of the Volatile Fraction of *Polygonum Bistorta* L. at Different Growing Stages and Evaluation of Its Antimicrobial Activity against Two Major Honeybee (*Apis Mellifera*) Pathogens. *Chem Biodivers* **2012**, *9*, 359–369, doi:10.1002/cbdv.201100326.

49. Osterc, G.; Petkovsek, M.M.; Stampar, F.; Kiprovski, B.; Ravnjak, B.; Bavcon, J. Characterization of Various Color Parameters (Anthocyanins and Flavonols) of Leaves and Flowers in Different Autochthonous Genotypes of *Cyclamen Purpurascens*. *Journal of the American Society for Horticultural Science* **2018**, *143*, 118–129, doi:10.21273/JASHS04320-17.

50. Moohammadnor, M.; Tursun, X.; Ling, M.Q.; Sultan, A.; Eshbakova, K.. A. Flavonoids from *Salix Caprea*. *Chem Nat Compd* **2010**, *46*, 799–800.

51. Sowndhararajan, K.; Kim, J.-H.; Song, J.E.; Kim, M.; Kim, S. Chemical Components of Male and Female Flowers of *Schisandra Chinensis*. *Biochem Syst Ecol* **2020**, *92*, 1–6, doi:10.1016/j.bse.2020.104121.

52. Yang, R.; Guan, Y.; Wang, W.; Chen, H.; He, Z.; Jia, A.Q. Antioxidant Capacity of Phenolics in *Camellia Nitidissima* Chi Flowers and Their Identification by HPLC Triple TOF MS/MS. *PLoS One* **2018**, *13*, 1–20, doi:10.1371/journal.pone.0195508.

53. Mohan Ram, H.Y.; Mathur, G. Flower Colour Changes in *Lantana Camara*. *J Exp Bot* **1984**, *35*, 1656–1662.

54. Fuselli, S.R.; de la Rosa, S.B.G.; Gende, L.B.; Eguaras, M.J.; Fritz, R. Antimicrobial Activity of Some Argentinean Wild Plant Essential Oils against *Paenibacillus Larvae Larvae*, Causal Agent of American Foulbrood (AFB). *J Apic Res* **2006**, *45*, 2–7, doi:10.1080/00218839.2006.11101304.

55. Fuselli, S.R.; Garcia de la Rosa, S.B.; Eguaras, M.J.; Fritz, R.; Ndagijimana, M.; Vannini, L.; Guerzoni, M.E. Efficacy of Indigenous Plant Essential Oil Andean Thyme (*Acantholippia Seriphioides* A. Gray) to Control American Foulbrood (AFB) in Honey Bee (*Apis Mellifera* L.) Hives. *Journal of Essential Oil Research* **2007**, *19*, 514–519, doi:10.1080/10412905.2007.9699319.

56. Bakour, M.; da Graça Campos, M.; Imtara, H.; Lyoussi, B. Antioxidant Content and Identification of Phenolic/Flavonoid Compounds in the Pollen of Fourteen Plants Using HPLC-DAD. *J Apic Res* **2020**, *59*, 35–41, doi:10.1080/00218839.2019.1675336.

57. Hisamichi, S. Microchemical Investigation on the Distribution of Pollen Flavonoid Components. *Yakugaku Zasshi* **1961**, *81*, 446–452.

58. Verónica C., S.; María De Los Ángeles, F.; Claudio R., G.; Fernanda, S.M. Analysis of Phenolic Compounds in Onion Nectar by Miniaturized Off-Line Solid Phase Extraction-Capillary Zone Electrophoresis. *Analytical Methods* **2014**, *6*, 4878–4884, doi:10.1039/c4ay00240g.

59. Duru, M.E.; Cakir, A.; Kordali, S.; Zengin, H.; Harmandar, M.; Izumi, S.; Hirata, T. Chemical Composition and Antifungal Properties of Essential Oils of Three *Pistacia* Species. *Fitoterapia* **2003**, *74*, 170–176, doi:10.1016/S0367-326X(02)00318-0.

60. Watson, R.W.; Levitin, N. Resin-Rubber from Canadian Grown Plants IV. Analytical Study of Milkweed Pod Gum. *Can J Res* **1946**, *24*, 95–105.

61. Lee, A.; Sugiura, Y.; Cho, I.-H.; Setou, N.; Koh, E.; Song, G.J.; Lee, S.; Yang, H.-J. In Vivo Hypoglycemic Effects, Potential Mechanisms and LC-MS/MS Analysis of *Dendropanax Trifidus* Sap Extract. *Nutrients* **2021**, *13*, 1–17, doi:10.3390/nu13124332.

62. Manning, R. Fatty Acids in Pollen: A Review of Their Importance for Honey Bees. *Bee World* **2001**, *82*, 60–75, doi:10.1080/0005772X.2001.11099504.

63. Drescher, N.; Wallace, H.M.; Katouli, M.; Massaro, C.F.; Leonhardt, S.D. Diversity Matters: How Bees Benefit from Different Resin Sources. *Oecologia* **2014**, *176*, 943–953, doi:10.1007/s00442-014-3070-z.

64. Borse, B.B.; Rao, L.J.M.; Ramalakshmi, K.; Raghavan, B. Chemical Composition of Volatiles from Coconut Sap (Neera) and Effect of Processing. *Food Chem* **2007**, *101*, 877–880, doi:10.1016/j.foodchem.2006.02.026.

65. Hebbar, K.B.; Arivalagan, M.; Pavithra, K.C.; Roy, T.K.; Gopal, M.; Shivashankara, K.S.; Chowdappa, P. Nutritional Profiling of Coconut (*Cocos Nucifera* L.) Inflorescence Sap Collected Using Novel Coco-Sap Chiller Method and Its Value Added Products. *Journal of Food Measurement and Characterization* **2020**, *14*, 2703–2712, doi:10.1007/s11694-020-00516-y.

66. Opute, F.I. Lipid and Sterol Composition of the Pollen of the West African Oil Palm, *Elaeis Guineensis*. *Phytochemistry* **1975**, *14*, 1023–1033.

67. Abed El Azim, M.H.M. Identification Phenolic and Biological Activities of Methanolic Extract of Date Palm Pollen (*Phoenix Dactylifera*). *J Microb Biochem Technol* **2015**, *07*, doi:10.4172/1948-5948.1000180.

68. Abu-Reidah, I.M.; Gil-Izquierdo, Á.; Medina, S.; Ferreres, F. Phenolic Composition Profiling of Different Edible Parts and By-Products of Date Palm (*Phoenix Dactylifera* L.) by Using HPLC-DAD-ESI/MSn. *Food Research International* **2017**, *100*, 494–500, doi:10.1016/j.foodres.2016.10.018.

69. Bentrad, N.; Gaceb-Terrak, R.; Rahmania, F. Identification and Evaluation of Antibacterial Agents Present in Lipophilic Fractions Isolated from Sub-Products of *Phoenix Dactilyfera*. *Nat Prod Res* **2017**, *31*, 2544–2548, doi:10.1080/14786419.2017.1314282.

70. Bentrad, N.; Gaceb-Terrak, R.; Benmalek, Y.; Rahmania, F. Studies on Chemical Composition and Antimicrobial Activities of Bioactive Molecules from Date Palm (*Phoenix Dactylifera* L.) Pollens and Seeds. *Afr J Tradit Complement Altern Med* **2017**, *14*, 242–256, doi:10.21010/ajtcam.v14i3.26.

71. Daoud, A.; Malika, D.; Bakari, S.; Hfaiedh, N.; Mnafgui, K.; Kadri, A.; Gharsallah, N. Assessment of Polyphenol Composition, Antioxidant and Antimicrobial Properties of Various Extracts of Date Palm Pollen (DPP) from Two Tunisian Cultivars. *Arabian Journal of Chemistry* **2019**, *12*, 3075–3086, doi:10.1016/j.arabjc.2015.07.014.

72. Salhi, S.; Chentouf, M.; Harrak, H.; Rahim, A.; Çakir, C.; Çam, D.; Öztürk, M.; Hamidallah, N.; Cabaraux, J.F.; El Amiri, B. Assessment of Physicochemical Parameters, Bioactive Compounds, Biological Activities, and Nutritional Value of the Most Two Commercialized Pollen Types of Date Palm (*Phoenix Dactylifera* L.) in Morocco. *Food Science and Technology International* **2023**, doi:10.1177/10820132231168914.

73. Ridi, M.S. El; Strait', L.A.; Aboul Wafa, M.H. Isolation of Rutin from the Pollen Grain of the Date Palm (*Dactylifera Palma* L.). *Arch Biochem Biophys* **1952**, *39*, 317–321.

74. Nicolson, S.W.; Human, H. Chemical Composition of the "low Quality" Pollen of Sunflower (*Helianthus Annuus*, Asteraceae). *Apidologie* **2013**, *44*, 144–152, doi:10.1007/s13592-012-0166-5.

75. Giacomini, J.J.; Leslie, J.; Tarpy, D.R.; Palmer-Young, E.C.; Irwin, R.E.; Adler, L.S. Medicinal Value of Sunflower Pollen against Bee Pathogens. *Sci Rep* **2018**, *8*, 1–10, doi:10.1038/s41598-018-32681-y.

76. LoCascio, G.M.; Aguirre, L.; Irwin, R.E.; Adler, L.S. Pollen from Multiple Sunflower Cultivars and Species Reduces a Common Bumblebee Gut Pathogen. *R Soc Open Sci* **2019**, *6*, 1–9, doi:10.1098/rsos.190279.

77. LoCascio, G.M.; Pasquale, R.; Amponsah, E.; Irwin, R.E.; Adler, L.S. Effect of Timing and Exposure of Sunflower Pollen on a Common Gut Pathogen of Bumble Bees. *Ecol Entomol* **2019**, *44*, 702–710, doi:10.1111/een.12751.

78. Adler, L.S.; Fowler, A.E.; Malfi, R.L.; Anderson, P.R.; Coppinger, L.M.; Deneen, P.M.; Lopez, S.; Irwin, R.E.; Farrell, I.W.; Stevenson, P.C. Assessing Chemical Mechanisms Underlying the Effects of Sunflower Pollen on a Gut Pathogen in Bumble Bees. *J Chem Ecol* **2020**, *46*, 649–658, doi:10.1007/s10886-020-01168-4.

79. Giacomini, J.J.; Connon, S.J.; Marulanda, D.; Adler, L.S.; Irwin, R.E. The Costs and Benefits of Sunflower Pollen Diet on Bumble Bee Colony Disease and Health. *Ecosphere* **2021**, *12*, 1–21, doi:10.1002/ecs2.3663.

80. Fowler, A.E.; Sadd, B.M.; Bassingthwaite, T.; Irwin, R.E.; Adler, L.S. Consuming Sunflower Pollen Reduced Pathogen Infection but Did Not Alter Measures of Immunity in Bumblebees. *Philosophical Transactions of the Royal Society B: Biological Sciences* **2022**, *377*, 1–8, doi:10.1098/rstb.2021.0160.

81. Fowler, A.E.; Kola, E.; Adler, L.S. The Effect of Sunflower Pollen Age and Origin on Pathogen Infection in the Common Eastern Bumble Bee (Apidae: Hymenoptera). *J Econ Entomol* **2023**, *116*, 1939–1942, doi:10.1093/jee/toad154.

82. Giacomini, J.J.; Adler, L.S.; Reading, B.J.; Irwin, R.E. Differential Bumble Bee Gene Expression Associated with Pathogen Infection and Pollen Diet. *BMC Genomics* **2023**, *24*, 1–18, doi:10.1186/s12864-023-09143-5.

83. Malfi, R.L.; McFrederick, Q.S.; Lozano, G.; Irwin, R.E.; Adler, L.S. Sunflower Plantings Reduce a Common Gut Pathogen and Increase Queen Production in Common Eastern Bumblebee Colonies. *Proceedings of the Royal Society B: Biological Sciences* **2023**, *290*, 1–10, doi:10.1098/rspb.2023.0055.

84. Palmer-Young, E.C.; Malfi, R.; Zhou, Y.; Joyce, B.; Whitehead, H.; Van Wyk, J.I.; Baylis, K.; Grubbs, K.; Boncristiani, D.L.; Evans, J.D.; et al. Sunflower-Associated Reductions in Varroa Mite Infestation of Honey Bee Colonies. *J Econ Entomol* **2023**, *116*, 68–77, doi:10.1093/jee/toac196.

85. Kolesnikov, M.P.; Gins, V.K. Flavonoids and Silicon in Certain Plant Pollen. *Chem Nat Compd* **1999**, *35*, 520–523.

86. Proksch, P.; Proksch', M.; Rundel, P.W.; Rodriguez, E. Ecological Significance of the Chemistry of the Leaf Resin of *Elytropappus Rhinocerotis*. *Biochem Syst Ecol* **1982**, *10*, 49–53.

87. Salonen, A.; Lavola, A.; Virjamo, V.; Julkunen-Tiitto, R. Protein and Phenolic Content and Antioxidant Capacity of Honey Bee-Collected Unifloral Pollen Pellets from Finland. *J Apic Res* **2021**, *60*, 744–750, doi:10.1080/00218839.2021.1902145.

88. Pratviel-Sosa, F.; Percheron, F. Les Sophorosides de Flavonols de Quelques Pollens. *Phytochemistry* **1972**, *11*, 1809–1813.

89. Meurer, B.; Wray, V.; Wiermann, R.; Strack, D. Hydroxycinnamic Acid-Spermidine Amides from Pollen of *Alnus Glutinosa*, *Betula Verrucosa*, *Pterocarya Fraxinifolia*. *Phytochemistry* **1988**, *27*, 839–843.

90. Dimkić, I.; Ristivojević, P.; Janakiev, T.; Berić, T.; Trifković, J.; Milojković-Opsenica, D.; Stanković, S. Phenolic Profiles and Antimicrobial Activity of Various Plant Resins as Potential Botanical Sources of Serbian Propolis. *Ind Crops Prod* **2016**, *94*, 856–871, doi:10.1016/j.indcrop.2016.09.065.

91. Meurer, B.; Wray, V.; Grotjahn, L.; Wiermann, R.; Strack, D. Hydroxycinnamic Acid Spermidine Amides from Pollen of *Corylus Avellana* L. *Phytochemistry* **1986**, *25*, 433–435.

92. Evans, D.E.; Rothnie, N.E.; Palmer, M. V.; Burke, D.G.; Sang, J.P.; Knox, R.B.; Williams, E.G.; Hilliard, E.P.; Salisbury, P.A. Comparative Analysis of Fatty Acids in Pollen and Seed of Rapseed. *Phytochemistry* **1987**, *26*, 1895–1897.

93. Singh, S.; Sawhney, V.K. Plant Hormones in *Brassica Napus* and *Lycopersicon Esculentum* Pollen. *Phytochemistry* **1992**, *31*, 4051–4053.

94. Jourdren, C.; Simonneaux, D.; Renard, M. Selection on Pollen for Linolenic Acid Content in Rapeseed, *Brassica Napus* L. *Plant Breeding* **1996**, *115*, 11–15, doi:10.1111/j.1439-0523.1996.tb00863.x.

95. Pei, D.; Liu, D.-H.; Liu, J.-X.; Di, D.-L. Chemical Investigation on the Pollen of *Brassica Napus*. *Chem Nat Compd* **2012**, *48*, 310–312.

96. Chen, X.; Pei, D.; Huang, X.Y.; Feng, Z.; Di, D. Effect of Ionic Liquids on Preparative Separation of Flavonoid Compounds in the Extract from *Brassica Napus* L. Pollen Using High-Performance Counter-Current Chromatography. *Sep Sci Technol* **2013**, *48*, 2890–2899, doi:10.1080/01496395.2013.804558.

97. Jin, L.; Liu, H.; Liu, X.; Gu, K.; Wu, M.; Huang, J. Efficient Production of Antioxidants from Rape Pollen via a Chromatographic Strategy. *Separations* **2022**, *9*, 1–9, doi:10.3390/separations9120445.

98. Ali, N.A.A.; Wurster, M.; Arnold, N.; Teichert, A.; Schmidt, J.; Lindequist, U.; Wessjohann, L. Chemical Composition and Biological Activities of Essential Oils from the Oleogum Resins of Three Endemic Soqotraen *Boswellia* Species. *Records of Natural Products* **2008**, *2*, 6–12.

99. Johnson, S.; DeCarlo, A.; Satyal, P.; Dosoky, N.S.; Sorensen, A.; Setzer, W.N. The Chemical Composition of Single-Tree *Boswellia Frereana* Resin Samples. *Nat Prod Commun* **2021**, *16*, doi:10.1177/1934578X211043727.

100. Gupta, M.; Rout, P.K.; Misra, L.N.; Gupta, P.; Singh, N.; Darokar, M.P.; Saikia, D.; Singh, S.C.; Bhakuni, R.S. Chemical Composition and Bioactivity of *Boswellia Serrata* Roxb. Essential Oil in Relation to Geographical Variation. *Plant Biosyst* **2017**, *151*, 623–629, doi:10.1080/11263504.2016.1187681.

101. Ayub, M.A.; Hanif, M.A.; Blanchfield, J.; Zubair, M.; Abid, M.A.; Saleh, M.T. Chemical Composition and Antimicrobial Activity of *Boswellia Serrata* Oleo-Gum-Resin Essential Oil Extracted by Superheated Steam. *Nat Prod Res* **2023**, *37*, 2451–2456, doi:10.1080/14786419.2022.2044327.

102. Abbas, R.K.; Al-Mushhin, A.A.M.; Elsharbasy, F.S.; Ashiry, K.O. Nutritive Value, Polyphenol Constituents and Prevention of Pathogenic Microorganism by Different Resin Extract of *Commiphora Myrrh*. *J Pure Appl Microbiol* **2020**, *14*, 1871–1878, doi:10.22207/JPAM.14.3.26.

103. Koudou, J.; Edou, P.; Obame, L.C.; Bassolé, I.H.; Figueredo, G. Volatile Components Antioxidants and Antimicrobial Properties of the Essential Oil of *Dacryodes Edulis* G. Don from Gabon. *Journal of Applied Sciences* **2008**, *8*, 3532–3535.

104. Bentes Machado, L.; Zoghbi, M. das G.B.; Andrade, E.H.A. Seasonal Variation in the Composition of the Essential Oils from the Leaves, Thin Branches and Resin of *Protium Spruceanum* (Benth.) Engl. *Flavour Fragr J* **2003**, *18*, 338–341, doi:10.1002/ffj.1238.

105. Zoghbi, M. das G.B.; Andrade, E.H.A.; Lima, M. da P.; Silva, T.M.D.; Daly, D.C. The Essential Oils of Five Species of Protium Growing in the North of Brazil. *Journal of Essential Oil-Bearing Plants* **2005**, *8*, 312–317, doi:10.1080/0972060X.2005.10643458.

106. Suárez, A.I.; Compagnone, R.S.; Acosta, D.; Vásquez, L.; Diaz, B.; Canelón, D.J. Chemical Composition and Antimicrobial Activity of the Essential Oil from Oleoresin of *Protium Neglectum* S. *Journal of Essential Oil-Bearing Plants* **2007**, *10*, 70–75, doi:10.1080/0972060X.2007.10643521.

107. Siani, A.C.; Ramos, M.F.S.; Monteiro, S. da S.; Ribeiro-dos-Santos, R.; Soares, R.O.A. Essential Oils of the Oleoresins from *Protium Heptaphyllum* Growing in the Brazilian Southeastern and Their Cytotoxicity to Neoplasic Cells Lines. *Journal of Essential Oil-Bearing Plants* **2011**, *14*, 373–378, doi:10.1080/0972060X.2011.10643948.

108. Ross, S.A.; ElSholy, M.A.; Sultana, G.N.N.; Mehmedic, Z.; Hossain, C.F.; Chandra, S. Flavonoid Glycosides and Cannabinoids from the Pollen of *Cannavis Sativa* L. *Phytochemical Analysis* **2005**, *16*, 45–48, doi:10.1002.pca.809.

109. Dingha, B.N.; Jackai, L.E. Chemical Composition of Four Industrial Hemp (*Cannabis Sativa* L.) Pollen and Bee Preference. *Insects* **2023**, *14*, 1–17, doi:10.3390/insects14080668.

110. Imperato, F. Five Plants of the Family Cucurbitaceae with Flavonoid Patterns of Pollens Different from Those of Corresponding Stigmas. *Experientia* **1980**, *36*, 1136–1137.

111. Ohmoto, T.; Yamaguchi, K. Constituents of Pollen. XV. Constituents of *Biota Orientalis* (L.) Endl. *Chem Pharm Bull (Tokyo)* **1988**, *36*, 807–809.

112. Silva, A.R.; Fernandes, Â.; García, P.A.; Barros, L.; Ferreira, I.C.F.R. *Cytinus Hypocistis* (L.) L. SubSp. *Macranthus* Wettst.: Nutritional Characterization. *Molecules* **2019**, *24*, doi:10.3390/molecules24061111.

113. Ferreres, F.; Andrade, P.; Gil, M.I.; Tomfis-Barberfin, F.A. Floral Nectar Phenolics as Biochemical Markers for the Botanical Origin of Heather Honey. *Z Lebensm Unters Forsch* **1996**, *202*, 40–44.

114. Koch, H.; Woodward, J.; Langat, M.K.; Brown, M.J.F.; Stevenson, P.C. Flagellum Removal by a Nectar Metabolite Inhibits Infectivity of a Bumblebee Parasite. *Current Biology* **2019**, *29*, 3494–3500, doi:10.1016/j.cub.2019.08.037.

115. Tourbez, C.; Semay, I.; Michel, A.; Michez, D.; Gerbaux, P.; Gekière, A.; Vanderplanck, M. Heather Pollen Is Not Necessarily a Healthy Diet for Bumble Bees. *Belg J Zool* **2023**, *153*, 105–124, doi:10.26496/bjz.2023.111.

116. Deiana, V.; Tuberoso, C.; Satta, A.; Pinna, C.; Camarda, I.; Spano, N.; Ciulu, M.; Floris, I. Relationship between Markers of Botanical Origin in Nectar and Honey of the Strawberry Tree (*Arbutus Unedo*) throughout Flowering Periods in Different Years and in Different Geographical Areas. *J Apic Res* **2015**, *54*, 342–349, doi:10.1080/00218839.2016.1164540.

117. Ferreres, F.; Andrade, P.; Tomá S-Barberán, F.A. Natural Occurrence of Abscisic Acid in Heather Honey and Floral Nectar. *J Agric Food Chem* **1996**, *44*, 2053–2056.

118. Egan, P.A.; Adler, L.S.; Irwin, R.E.; Farrell, I.W.; Palmer-Young, E.C.; Stevenson, P.C. Crop Domestication Alters Floral Reward Chemistry with Potential Consequences for Pollinator Health. *Front Plant Sci* **2018**, *9*, 1–14, doi:10.3389/fpls.2018.01357.

119. Guo, F.; Yang, Y.; Duan, Y.; Li, C.; Gao, H.; Liu, H.; Cui, Q.; Guo, Z.; Liu, X.; Wang, Z. Quality Marker Discovery and Quality Evaluation of *Eucommia Ulmoides* Pollen Using UPLC-QTOF-MS Combined with a DPPH-HPLC Antioxidant Activity Screening Method. *Molecules* **2023**, *28*, doi:10.3390/molecules28135288.

120. Folly, A.J.; Koch, H.; Farrell, I.W.; Stevenson, P.C.; Brown, M.J.F. Agri-Environment Scheme Nectar Chemistry Can Suppress the Social Epidemiology of Parasites in an Important Pollinator. *Proceedings of the Royal Society B: Biological Sciences* **2021**, *288*, 1–10, doi:10.1098/rspb.2021.0363.

121. Irwin, R.E.; Adler, L.S. Correlations among Traits Associated with Herbivore Resistance and Pollination: Implications for Pollination and Nectar Robbing in a Distylous Plant. *Am J Bot* **2006**, *93*, 64–72, doi:10.3732/ajb.93.1.64.

122. Qiu, J.; Chen, X.; Netrusov, A.I.; Zhou, Q.; Guo, D.; Liu, X.; He, H.; Xin, X.; Wang, Y.; Chen, L. Screening and Identifying Antioxidative Components in *Ginkgo Biloba* Pollen by DPPHHPLC-PAD Coupled with HPLC-ESI-MS2. *PLoS One* **2017**, *12*, doi:10.1371/journal.pone.0170141.

123. Żurek, N.; Pycia, K.; Pawłowska, A.; Kapusta, I.T. Phytochemical Screening and Bioactive Properties of *Juglans Regia* L. Pollen. *Antioxidants* **2022**, *11*, 1–12, doi:10.3390/antiox11102046.

124. Wiese, N.; Fischer, J.; Heidler, J.; Lewkowski, O.; Degenhardt, J.; Erler, S. The Terpenes of Leaves, Pollen, and Nectar of Thyme (*Thymus Vulgaris*) Inhibit Growth of Bee Disease-Associated Microbes. *Sci Rep* **2018**, *8*, 1–12, doi:10.1038/s41598-018-32849-6.

125. Togasawa, Y.; Katsumata, T.; Kawajiri, H.; Onodera, N. Biochemical Studies on Pollen V. Flavonoid Pigments from Pollen of *Lilium Lancifolium* Thunb. *Nippon Nogeikagaku Kaishi* **1966**, *40*, 461–465.

126. Togasawa, Y.; Katsumata, T.; Fukada, M.; Motoi, T. Biochemical Studies on Pollen Part VII. Vitamins of Pollen. *Nippon Nogeikagaku Kaishi* **1967**, *41*, 184–188.

127. Kleinehollenhorst, G.; Behrens, H.; Pegels, G.; Srunk, N.; Wiermann, R. Formation of Flavonol 3-O-Diglycosides and Flavonol 3-O-Trigly-Cosides by Enzyme Extracts from Anthers of *Tulipa* Cv. Apeldoorn: Characterization and Activity of Three Different O-Glycosyltransferases during Anther Development. *Zeitschrift für Naturforschung C* **1982**, *37*, 587–599.

128. Naef, R.; Jaquier, A.; Velluz, A.; Bachofen, B. From the Linden Flower to Linden Honey - Volatile Constituents of Linden Nectar, the Extract of Bee-Stomach and Ripe Honey. *Chem Biodivers* **2004**, *1*, 1870–1879, doi:10.1002/cbdv.200490143.

129. Gašić, U.; Šikoparija, B.; Tosti, T.; Trifković, J.; Milojković-Opsenica, D.; Natić, M.; Tešić, Ž. Phytochemical Fingerprints of Lime Honey Collected in Serbia. *J AOAC Int* **2014**, *97*, 1259–1267, doi:10.5740/jaoacint.SGEGasic.

130. Lande, C.; Rao, S.; Morré, J.T.; Galindo, G.; Kirby, J.; Reardon, P.N.; Bobe, G.; Stevens, J.F. Linden (*Tilia Cordata*) Associated Bumble Bee Mortality: Metabolomic Analysis of Nectar and Bee Muscle. *PLoS One* **2019**, *14*, 1–16, doi:10.1371/journal.pone.0218406.

131. Chansriniyom, C.; Nooin, R.; Nuengchamnong, N.; Wongwanakul, R.; Petpiroon, N.; Srinuanchai, W.; Chantarasuwan, B.; Pitchakarn, P.; Temviriyanukul, P.; Nuchuchua, O. Tandem Mass Spectrometry of Aqueous Extract from Ficus Dubia Sap and Its Cell-Based Assessments for Use as a Skin Antioxidant. *Sci Rep* **2021**, *11*, doi:10.1038/s41598-021-96261-3.

132. Lee, S.W.; Hung, W.J.; Chen, Z.T. A New Flavonol from the Kino of *Eucalyptus Citriodora*. *Nat Prod Res* **2017**, *31*, 37–42, doi:10.1080/14786419.2016.1209667.

133. Lee, S.-W. A New Lipoxygenase Inhibitory Flavonoid from the Kino of *Eucalyptus Citriodora*. *Chem Nat Compd* **2019**, *55*, 18–20, doi:10.1007/s10600-019-02655-1.

134. Ali, D.E.; El Gedaily, R.A.; Ezzat, S.M.; El Sawy, M.A.; Meselhy, M.R.; Abdel-Sattar, E. In Silico and in Vitro Anti-Inflammatory Study of Phenolic Compounds Isolated from *Eucalyptus Maculata* Resin. *Sci Rep* **2023**, *13*, 1–12, doi:10.1038/s41598-023-28221-y.

135. Wollenweber, E.; Wiermann, R. On the Pigmentation of the Pollen of *Nothofagus Antarctica* (Forst.) Oerst. (Fagaceae). *Zeitschrift für naturforschung* **1979**, *34*, 1289–1291.

136. Fujita, M.; Hisamichi, S.; Ando, T.; Murakami, N. Investigations on Flavonoid Component in the Pollen of Some *Forsythia* Species. *Chem Pharm Bull (Tokyo)* **1960**, *8*, 1124–1127.

137. Al-Dehadheh, A.M.; Qrunfleh, M.M.; Ateyyeh, A.F. Morphology, Viability, in Vitro Germination and Auxin Content of Pollen of Five Olive (*Olea Europaea* L.) Cultivars. *Adv Hortic Sci* **2004**, *18*, 68–73.

138. Netlak, P.; Imsabai, W.; Munne-Bosch, S.; Leethiti, P.; Van Doorn, W.G. Identification of Indole-3-Acetic Acid as an Important Hormone in Post- Pollination of *Dendrobium* Orchids and Interaction of Other Hormones. *Agriculture and Natural Resources* **2022**, *56*, 149–158, doi:10.34044/j.anres.2021.56.1.14.

139. Karioti, A.; Kitsaki, C.K.; Zygouraki, S.; Ziobora, M.; Djeddi, S.; Skaltsa, H.; Liakopoulos, G. Occurrence of Flavonoids in *Ophrys* (Orchidaceae) Flower Parts. *Flora: Morphology, Distribution, Functional Ecology of Plants* **2008**, *203*, 602–609, doi:10.1016/j.flora.2007.09.009.

140. Ching, T.M.; Ching, K.K. Fatty Acids in Pollen of Some Coniferous Species. *Science (1979)* **1962**, *138*, 890–891.

141. Scott, R.W.; Strohl, M.J. Extraction and Identification of Lipids from Loblolly Pine Pollen. *Phytochemistry* **1962**, *1*, 189–193.

142. Strohl, M.J.; Seikel, M.K. Polyphenols of Pine Pollens. *Phytochemistry* **1965**, *4*, 383–399.

143. Shibuya, T.; Funamizu, M.; Kitahara, Y. Abscisic Acid from *Pinus Densiflora* Pollen. *Phytochemistry* **1978**, *17*, 322–323.

144. Khan, V.A.; Bol'shakova, V.I.; Grigorovich, M.I.; Shmidt, É.N.; Dubovenko, Zh. V.; Pentegova, V.A. Terpenoids of *Pinus Funebris*. *Chem Nat Compd* **1984**, *20*, 420–423.

145. Ohmoto, T.; Kanatani, K.; Yamaguchi, K. Constituent of Pollen. XIII. Constituents of *Cedrus Deodara* Loud. (2). *Chem Pharm Bull (Tokyo)* **1987**, *35*, 229–234.

146. Richardson, L.L.; Bowers, M.D.; Irwin, R.E. Nectar Chemistry Mediates the Behavior of Parasitized Bees: Consequences for Plant Fitness. *Ecology* **2016**, *97*, 325–337, doi:10.1890/15-0263.1.

147. Ceska, O.; Styles, E.D. Flavanoids from *Zea Mays* Pollen. *Phytochemistry* **1984**, *23*, 1822–1823.

148. Bianchi, G.; Murelli, C.; Ottaviano, E. Maize Pollen Lipids. *Phytochemistry* **1990**, *29*, 739–744.

149. Žilić, S.; Vančetović, J.; Janković, M.; Maksimović, V. Chemical Composition, Bioactive Compounds, Antioxidant Capacity and Stability of Floral Maize (*Zea Mays* L.) Pollen. *J Funct Foods* **2014**, *10*, 65–74, doi:10.1016/j.jff.2014.05.007.

150. Kostić, A.; Mačukanović-Jocić, M.P.; Špirović Trifunović, B.D.; Vukašinović, I.; Pavlović, V.B.; Pešić, M.B. Fatty Acids of Maize Pollen – Quantification, Nutritional and Morphological Evaluation. *J Cereal Sci* **2017**, *77*, 180–185, doi:10.1016/j.jcs.2017.08.004.

151. Li, F.; Guo, S.; Zhang, S.; Peng, S.; Cao, W.; Ho, C.-T.; Bai, N. Bioactive Constituents of *F. Esculentum* Bee Pollen and Quantitative Analysis of Samples Collected from Seven Areas by HPLC. *Molecules* **2019**, *24*, 1–15, doi:10.3390/molecules24152705.

152. Nešović, M.; Gašić, U.; Tosti, T.; Horvacki, N.; Šikoparija, B.; Nedić, N.; Blagojević, S.; Ignjatović, L.; Tešić, Ž. Polyphenol Profile of Buckwheat Honey, Nectar and Pollen: Polyphenolics in Buckwheat. *R Soc Open Sci* **2020**, *7*, 1–15, doi:10.1098/rsos.201576.

153. Rutuparna, J.; Ali, A.; Ghazi, I.A. Comparative Metabolomic Analysis of Unreleased and Released Pollen from *Putranjiva Roxburghii* Wall. *South African Journal of Botany* **2022**, doi:10.1016/j.sajb.2022.07.017.

154. Ferreres, F.; Tomas-Barberan, F.A.; Tomas-Lorente, F.; Nieto, J.L.; Rumbero, A.; Olias, J.M. 8-Methoxykaempferol 3-Sophoroside, a Yellow Pigment from Almond Pollen. *Phytochemistry* **1989**, *28*, 1901–1903.

155. London-Shafir, I.; Shafir, S.; Eisikowitch, D. Amygdalin in Almond Nectar and Pollen - Facts and Possible Roles. *Plant Systematics and Evolution* **2003**, *238*, 87–95, doi:10.1007/s00606-003-0272-y.

156. McNulty, J.; Nair, J.J.; Bollareddy, E.; Keskar, K.; Thorat, A.; Crankshaw, D.J.; Holloway, A.C.; Khan, G.; Wright, G.D.; Ejim, L. Isolation of Flavonoids from the Heartwood and Resin of *Prunus Avium* and Some Preliminary Biological Investigations. *Phytochemistry* **2009**, *70*, 2040–2046, doi:10.1016/j.phytochem.2009.08.018.

157. Guffa, B.; Nedić, N.M.; Dabić Zagorac, D.C.; Tosti, T.B.; Gašić, U.M.; Natić, M.M.; Fotirić Akšić, M.M. Characterization of Sugar and Polyphenolic Diversity in Floral Nectar of Different 'Oblačinska' Sour Cherry Clones. *Chem Biodivers* **2017**, *14*, 1–15, doi:10.1002/cbdv.201700061.

158. Akšic, M.F.; Gašic, U.; Zagorac Dragana, D.; Sredojevic, M.; Tosti, T.; Natic, M.; Meland, M. Chemical Fingerprint of 'Oblacinska' Sour Cherry (*Prunus Cerasus* L.) Pollen. *Biomolecules* **2019**, *9*, doi:10.3390/biom9090391.

159. Bosco, R.; Caser, M.; Vanara, F.; Scariot, V. Development of a Rapid LC-DAD/FLD Method for the Simultaneous Determination of Auxins and Abscisic Acid in Plant Extracts. *J Agric Food Chem* **2013**, *61*, 10940–10947, doi:10.1021/jf4034305.

160. Wright, G.A.; Baker, D.D.; Palmer, M.J.; Stabler, D.; Mustard, J.A.; Power, E.F.; Borland, A.M.; Stevenson, P.C. Caffeine in Floral Nectar Enhances a Pollinator's Memory of Reward. *Science (1979)* **2013**, *339*, 1202–1204, doi:10.1126/science.1228806.

161. Kretschmar, J.A.; Baumann, T.W. Caffeine in Citrus Flowers. *Phytochemistry* **1999**, *52*, 19–23.

162. Huang, H.H.; Lin, L.Y.; Chiang, H.M.; Lay, S.J.; Wu, C.S.; Chen, H.C. Analysis of Volatile Compounds from Different Parts of *Citrus Grandis* (L.) Osbeck Flowers by Headspace Solid-Phase Microextraction-Gas Chromatography-Mass Spectrometry. *Journal of Essential Oil-Bearing Plants* **2017**, *20*, 1057–1065, doi:10.1080/0972060X.2017.1377112.

163. Palmer-Young, E.C.; Farrell, I.W.; Adler, L.S.; Milano, N.J.; Egan, P.A.; Junker, R.R.; Irwin, R.E.; Stevenson, P.C. Chemistry of Floral Rewards: Intra- and Interspecific Variability of Nectar and Pollen Secondary Metabolites across Taxa. *Ecol Monogr* **2019**, *89*, 1–23, doi:10.1002/ecm.1335.

164. Barascou, L.; Sene, D.; Barraud, A.; Michez, D.; Lefebvre, V.; Medrzycki, P.; Di Prisco, G.; Strobl, V.; Yañez, O.; Neumann, P.; et al. Pollen Nutrition Fosters Honeybee Tolerance to Pesticides. *R Soc Open Sci* **2021**, *8*, 1–15, doi:10.1098/rsos.210818.

165. Martinez, A.; Calhoun, A.C.; Sadd, B.M. Investigating the Influence of Diet Diversity on Infection Outcomes in a Bumble Bee (*Bombus Impatiens*) and Microsporidian (*Nosema Bombi*) Host-Pathogen System. *Frontiers in Insect Science* **2023**, *3*, 1–10, doi:10.3389/finsc.2023.1207058.

166. Paetz, C.; Hammerbacher, A.; Menezes, R.C.; Feistel, F.; Weigel, C.; Voigt, K.; Schneider, B. Chemical Composition and Antimicrobial Activity of *Populus Nigra* Shoot Resin. *Natural Product Coomunications* **2016**, *11*, 989–992.

167. Lagacé, L.; Camara, M.; Martin, N.; Ali, F.; Houde, J.; Corriveau, S.; Sadiki, M. Effect of the New High Vacuum Technology on the Chemical Composition of Maple Sap and Syrup. *Heliyon* **2019**, *5*, 1–6, doi:10.1016/j.heliyon.2019.e01786.

168. Tadmor-Melamed, H.; Markman, S.; Arieli, A.; Distl, M.; Wink, M.; Izhaki, I. Limited Ability of Palestine Sunbirds *Nectarinia Osea* to Cope with Pyridine Alkaloids in Nectar of Tree Tobacco *Nicotiana Glauca*. *Funct Ecol* **2004**, *18*, 844–850, doi:10.1111/j.0269-8463.2004.00929.x.

169. Adler, L.S.; Wink, M.; Distl, M.; Lentz, A.J. Leaf Herbivory and Nutrients Increase Nectar Alkaloids. *Ecol Lett* **2006**, *9*, 960–967, doi:10.1111/j.1461-0248.2006.00944.x.

170. Kessler, D.; Baldwin, I.T. Making Sense of Nectar Scents: The Effects of Nectar Secondary Metabolites on Floral Visitors of *Nicotiana Attenuata*. *The Plant Journal* **2006**, *49*, 840–854, doi:10.1111/J.1365-313X.2006.02995.X.

171. Kessler, D.; Bhattacharya, S.; Diezel, C.; Rothe, E.; Gase, K.; Schöttner, M.; Baldwin, I.T. Unpredictability of Nectar Nicotine Promotes Outcrossing by Hummingbirds in *Nicotiana Attenuata*. *Plant Journal* **2012**, *71*, 529–538, doi:10.1111/j.1365-313X.2012.05008.x.

172. Kerchner, A.; Darók, J.; Bacskay, I.; Felinger, A.; Jakab, G.; Farkas, Á. Protein and Alkaloid Patterns of the Floral Nectar in Some Solanaceous Species. *Acta Biol Hung* **2015**, *66*, 304–315, doi:10.1556/018.66.2015.3.6.

173. Heng, M.Y.; Syafni, N.; Ramseyer, J.; Thuerig, B.; Tamm, L.; Hamburger, M.; Potterat, O. Qualitative and Auantitative Secondary Metabolite Profiles in a Large Set of Sumatra Benzoin Samples. *J Agric Food Chem* **2023**, *71*, 10590–10597, doi:10.1021/acs.jafc.3c01861.

174. Sykut, A. Carotenoids in Indian Cress Flowers (*Tropaeolum Majus*). *Acta Societatis Botanicorum Poloniae* **1966**, *35*, 1–17.

175. Caffrey, M.; Werner, B.G.; Priestley, D.A. A Crystalline Lipid Phase in a Dry Biological System: Evidence from X-Ray Diffraction Analysis of *Typha Latifolia* Pollen. *Biochim Biophys Acta* **1987**, *921*, 124–134.

176. Tao, W.; Yang, N.; Duan, J.; Wu, D.; Guo, J.M.; Tang, Y.; Qian, D.; Zhu, Z. Simultaneous Determination of Eleven Major Flavonoids in the Pollen of *Typha Angustifolia* by HPLC-PDA-MS. *Phytochemical Analysis* **2011**, *22*, 455–461, doi:10.1002/pca.1302.

177. Han, L.; Liu, X.; Yang, N.; Li, J.; Cai, B.; Cheng, S. Simultaneous Chromatographic Fingerprinting and Quantitative Analysis of Flavonoids in Pollen Typhae by High-Performance Capillary Electrophoresis. *Acta Pharm Sin B* **2012**, *2*, 602–609, doi:10.1016/j.apsb.2012.10.003.

178. Meng, Z.; Zhao, J.; Duan, H.; Guan, Y.; Zhao, L. Green and Efficient Extraction of Four Bioactive Flavonoids from Pollen Typhae by Ultrasound-Assisted Deep Eutectic Solvents Extraction. *J Pharm Biomed Anal* **2018**, *161*, 246–253, doi:10.1016/j.jpba.2018.08.048.

179. Wang, X.; Li, J.; Yang, X.; Gao, X.; Wang, H.; Chang, Y. A Rapid and Efficient Extraction Method Based on Industrial MCM-41-Miniaturized Matrix Solid-Phase Dispersion Extraction with Response Surface Methodology for Simultaneous Quantification of Six Flavonoids in Pollen Typhae by Ultra-High-Performance Liquid Chromatography. *J Sep Sci* **2019**, *42*, 2426–2434, doi:10.1002/jssc.201900227.
